# Cortical Endogenic Neural Regeneration of Adult Rat after Traumatic Brain Injury

**DOI:** 10.1371/journal.pone.0070306

**Published:** 2013-07-29

**Authors:** Xin Yi, Guohua Jin, Xinhua Zhang, Weifeng Mao, Haoming Li, Jianbing Qin, Jinhong Shi, Kui Dai, Fan Zhang

**Affiliations:** 1 Department of Anatomy and Cytoneurobiology, Medical College of Soochow University, Suzhou, China; 2 Department of Anatomy and Neurobiology, The Jiangsu Key Laboratory of Neuroregeneration, Nantong University, Nantong, China; 3 Xinglin College of Nantong University, Nantong, China; Massachusetts General Hospital/Harvard Medical School, United States of America

## Abstract

Focal and diffuse neuronal loss happened after traumatic brain injury (TBI). With little in the way of effective repair, recent interest has focused on endogenic neural progenitor cells (NPCs) as a potential method for regeneration. Whether endogenic neural regeneration happened in the cortex of adult rat after TBI remains to be determined. In this study, rats were divided into a sham group and a TBI group, and the rat model of medium TBI was induced by controlled cortical impact. Rats were injected with BrdU at 1 to 7 days post-injury (dpi) to allow identification of differentiated cells and sacrificed at 1, 3, 7, 14 and 28 dpi for immunofluorescence. Results showed nestin^+^/sox-2^+^ NPCs and GFAP^+^/sox-2^+^ radial glial (RG)-like cells emerged in peri-injured cortex at 1, 3, 7, 14 dpi and peaked at 3 dpi. The number of GFAP^+^/sox-2^+^ cells was less than that of nestin^+^/sox-2^+^ cells. Nestin^+^/sox-2^+^ cells from posterior periventricle (pPV) immigrated into peri-injured cortex through corpus callosum (CC) were found. DCX^+^/BrdU^+^ newborn immature neurons in peri-injured cortex were found only at 3, 7, 14 dpi. A few MAP-2^+^/BrdU^+^ newborn neurons in peri-injured cortex were found only at 7 and 14 dpi. NeuN^+^/BrdU^+^ mature neurons were not found in peri-injured cortex at 1, 3, 7, 14 and 28 dpi. While GFAP^+^/BrdU^+^ astrocytes emerged in peri-injured cortex at 1, 3, 7, 14, 28 dpi and peaked at 7 dpi then kept in a stable state. In the corresponding time point, the percentage of GFAP^+^/BrdU^+^ astrocytes in BrdU^+^ cells was more than that of NPCs or newborn neurons. No CNP^+^/BrdU^+^ oligodendrocytes were found in peri-injured cortex. These findings suggest that NPCs from pPV and reactive RG–like cells emerge in peri-injured cortex of adult rats after TBI. It can differentiate into immature neurons and astrocytes, but the former fail to grow up to mature neurons.

## Introduction

Incidences of traumatic brain injury (TBI) in both developing and developed countries are in rise for growth in population and increasing number of traffic accidents. Moreover modern military conflict has led to an additional steep rise in TBI due to direct combat-mediated head injuries [1]. Not only primary insult result in local neuronal destruction, but also mechanical brain injury secondarily induces a progressive cascade of related events that result in neuronal death, including brain edema, ischemia, diffuse axonal injury, radical-mediated damage, excitotoxicity, mitochondrial dysfunction, and dysregulation of calcium homeostasis [2], [3]. Although an improved understanding of the pathophysiology that occurs in TBI, clinical neuroprotection trials pharmacologically targeting at preventing cellular death after TBI have failed to show consistent improvement in outcome for patients with TBI [4], [5]. So many survival head-injured patients were long-term disability for permanent neurological impairment. The economic and health burden of TBI is significant and is predicted to grow further in the next decade [6], [7].

With the lack of effective therapy to repair injured brain tissue, has motivated researchers to focus on stem cells as a potential avenue for regeneration. Much evidence of functional recovery was provided by the use of exogenous stem cells in TBI in rodent models [8]–[11]. However, there are still many questions to be answered, for instance, ethical and theoretical issues, the appropriate source of stem cells, immune rejection and so on. Recently, interest in promoting regeneration of the TBI has turned toward the use of endogenous stem cells.

Continual neurogenesis in the adult human subgranular zone (SGZ) of hippocampus [12] and subventricular zone (SVZ) [13], [14] is accepted and enhancement of neurogenesis in the two zones is confirmed in some rodent models with brain injury [15]–[18]. Whether adult neurogenesis is constrained only in the two canonical zones, or could occur also in other central nervous system (CNS) areas is still an open question [19]. It was largely accepted that the cerebral cortex of adults is non-neurogenic until a few years ago [20], [21]. Some researchers reported that stem cells from damaged adult rat cerebral cortex and adult human neocortex after TBI had been isolated in vitro [22], [23]. The origin of them (immigrating cells or the residing cells?) remains controversial. Recent studies have demonstrated that the posterior periventricle (pPV), which is the posterior part of the lateral ventricle (LV), also retains proliferating neural progenitor cells (NPCs) [24] and they could migrate into the hippocampal CA1 region in rats after ischemic injury [25]. Whether they migrate into peri-injured cortex after TBI is still unknown and further studies are needed to determine whether there are cells residing in the cortex that harbor the ability to regeneration on activation by injury.

In our current study, we used a controlled cortical impact (CCI) adult rat model of medium TBI, and observed whether endogenous neural regeneration happened in the adult rat cortex after TBI by immunofluorescence.

## Materials and Methods

### 2.1 Ethics Statement

All animal experiments were carried out in accordance with the United States National Institutes of Health Guide for the Care and Use of Laboratory Animals (NIH Publication No. 85-23, revised 1996). The study protocol was approved by the Care and Use committee of Laboratory Animal Research Center of Nantong University.

### 2.2 Rats Model of CCI Focal TBI

Sprague–Dawley (220–250 g) rats were purchased from the Experimental Animal Center of Nantong University. The rats were divided into 6 groups: 1 day post-injury (dpi) group, 3 dpi group, 7 dpi group, 14 dpi group, 28 dpi group and sham group. There were 6 rats in each group. After transient anesthesia with Chlorpent (2 ml/kg body weight), rats were transferred to the stereotaxic apparatus. Under aseptic conditions rats underwent craniotomy surgery to create a circular window (4 mm diameter) centered at antero-posterior (A/P) −3.5 mm, medio-lateral (M/L) 2.5 mm with reference to the bregma, taking care to avoid damaging the meninges. The bone flap was removed and rats were subjected to controlled cortical impact using a pneumatic impact device (Model FP302, AmScien Instruments LLC, USA) with a 1.5 atm (1 atm = 101.325 kPa) pressure. Immediately after generating the brain trauma the craniotomy hole was sealed with bone wax, the scalp incision was closed with sutures. Sham control rats were subjected to all aspects of the protocol (anesthesia, skin incision, and craniotomy) except for cortical impact. After recovery from anesthesia, the rats were returned to their cages with postoperative care and adlibitem access to food and water.

### 2.3 BrdU Injection

Rats were injected intraperitoneally with BrdU (50 mg/kg, twice a day) at 1 to 7 days post-injury (dpi) to label newborn neurons.

### 2.4 Immunohistochemistry

At the end of the appropriate follow-up period: 1, 3, 7, 14 and 28 dpi, the rats were deeply anesthetized and were perfusion fixed intracardially with 0.9% NaCl followed by 4% paraformaldehyde in 0.1 M phosphate buffer (PB). The brains were removed and post-fixed in 4% paraformaldehyde for 4 hours at room temperature (RT). Brain coronal sections of 16 µm thicknss (−2.5 to −3.5 mm from the bregma) were prepared using a cryostat (Leica CM1900), blocked in 10% goat serum in PBST (0.01 M sodium phosphate buffer, pH 7.4, containing 0.05% (v/v) Tween 20) for 1 h at RT. The sections were incubated with primary antibodies: rat anti-BrdU (1∶100, Abcam, UK. Newborn cells marker), mouse monoclonal anti-nestin (1∶2000, Millipore, USA. Stem cells marker), mouse monoclonal anti-GFAP (1∶200, Sigma. Astrocyte/glial marker), rabbit anti-sox-2 (1∶1000, Abcam. Stem cells marker), guinea pig anti-DCX (1∶1000, Millipore. Immature neurons marker), mouse monoclonal anti-MAP-2 (1∶500, Millipore. Semi-mature and mature neurons marker), mouse monoclonal anti-NeuN (1∶500, Millipore. Mature neurons marker), mouse monoclonal anti-CNP (1∶800, Millipore. Oligodendrocytes marker), overnight at 4°C, followed by incubation with Alexa Fluor 568-conjugated goat anti-rat or rabbit IgG (1∶1000, Molecular Probes), 488-conjugated goat anti-mouse or guinea pig IgG (1∶200, invitrogen).

### 2.5 Statistical Analysis

The immunopositive cells in the peri-injured cortex, corpus callosum (CC) and posterior periventricle (pPV) of three brain coronal sections (front, middle and rear injury zone) in each rat were counted by single blind method. Statistical analysis was performed using statistics package for social science 16.0 (SPSS 16.0). Statistical comparisons were performed using one-way analysis of variance (ANOVA) and differences at *p*<0.05 were considered statistically significant.

## Results

### 3.1 The Size of the Injury

**Figure 1:**
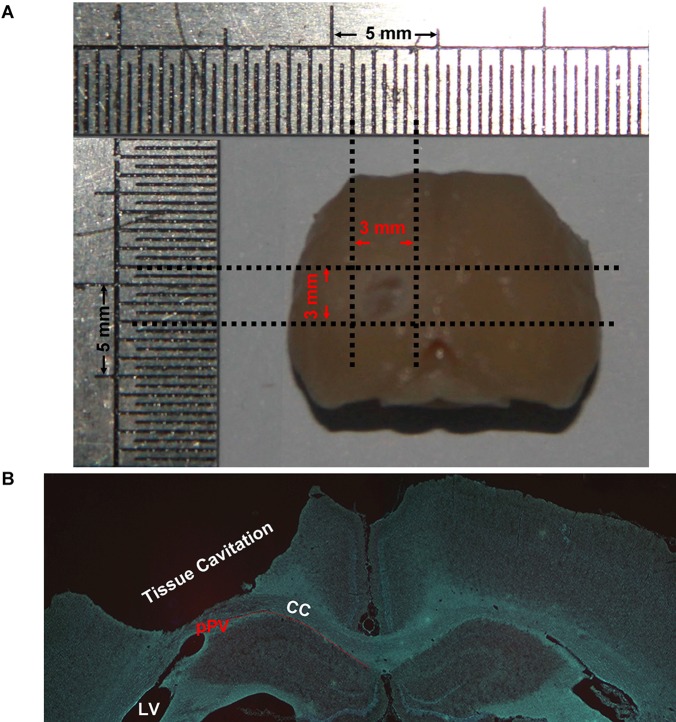
The size of the injury. After traumatic brain injury (TBI) there was a cylindrical tissue cavitation in the injury cortex. The size of the injury was 1.5±0.09 mm (dorso-ventral), 3±0.24 mm (medio-temporal) and 3±0.29 mm (rostro-caudal). (A) Macroscopical dorsal view of the brain after TBI. (B) A low magnification hoechst staining image of a coronal section −2.8 mm to the bregma.

Rat model of medium TBI was induced by controlled cortical impact. After TBI there was a cylindrical tissue cavitation in the injury cortex. The size of the injury was about 1.5 mm (dorso-ventral), 3 mm (medio-temporal) and 3 mm (rostro-caudal) (Figure 1).

### 3.2 Endogenic Nestin^+^/sox-2^+^ NPCs in the Peri-injured Cortex, CC and pPV

To confirm whether endogenous NPCs emerged in the adult rat cortex after TBI, the immunofluorescence of nestin/sox-2 and GFAP/sox-2 double staining was applied to detect the NPCs in the cortex, CC and pPV.

**Figure 2:**
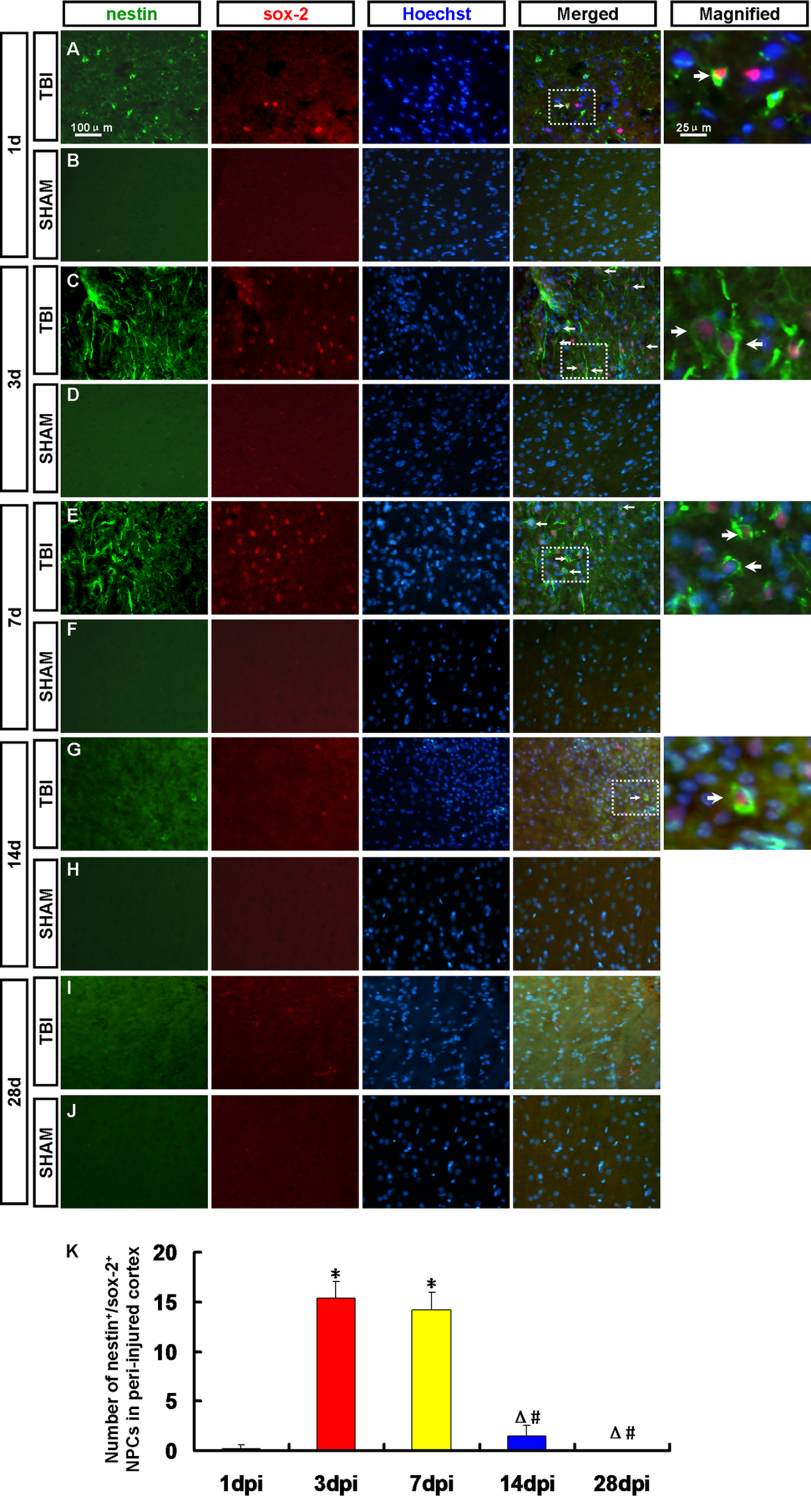
Endogenic nestin^+^/sox-2^+^ neural progenitor cells (NPCs) in the peri-injured cortex. NPCs were detected by nestin (green) and sox-2 (red) antibody. Arrows denote the nestin^+^/sox-2^+^ cells. (A, C, E, G, I) Nestin^+^/sox-2^+^ NPCs were observed in the peri-injured cortex at 1, 3, 7, 14 and 28 dpi. (B, D, F, H, J) No Nestin^+^/sox-2^+^ NPCs were found in the cortex of rats in the sham group. (K) A statistic diagram for the number of nestin^+^/sox-2^+^ NPCs in the peri-injured cortex at 1, 3, 7, 14 and 28 dpi. The number of Nestin^+^/sox-2^+^ NPCs peaked at 3 dpi then decreased. ^*^ *p*<0.05, vs. 1 dpi group; ^△^ *p*<0.05, vs. 3 dpi group; ^#^ *p*<0.05 vs. 7 dpi group.

**Figure 3:**
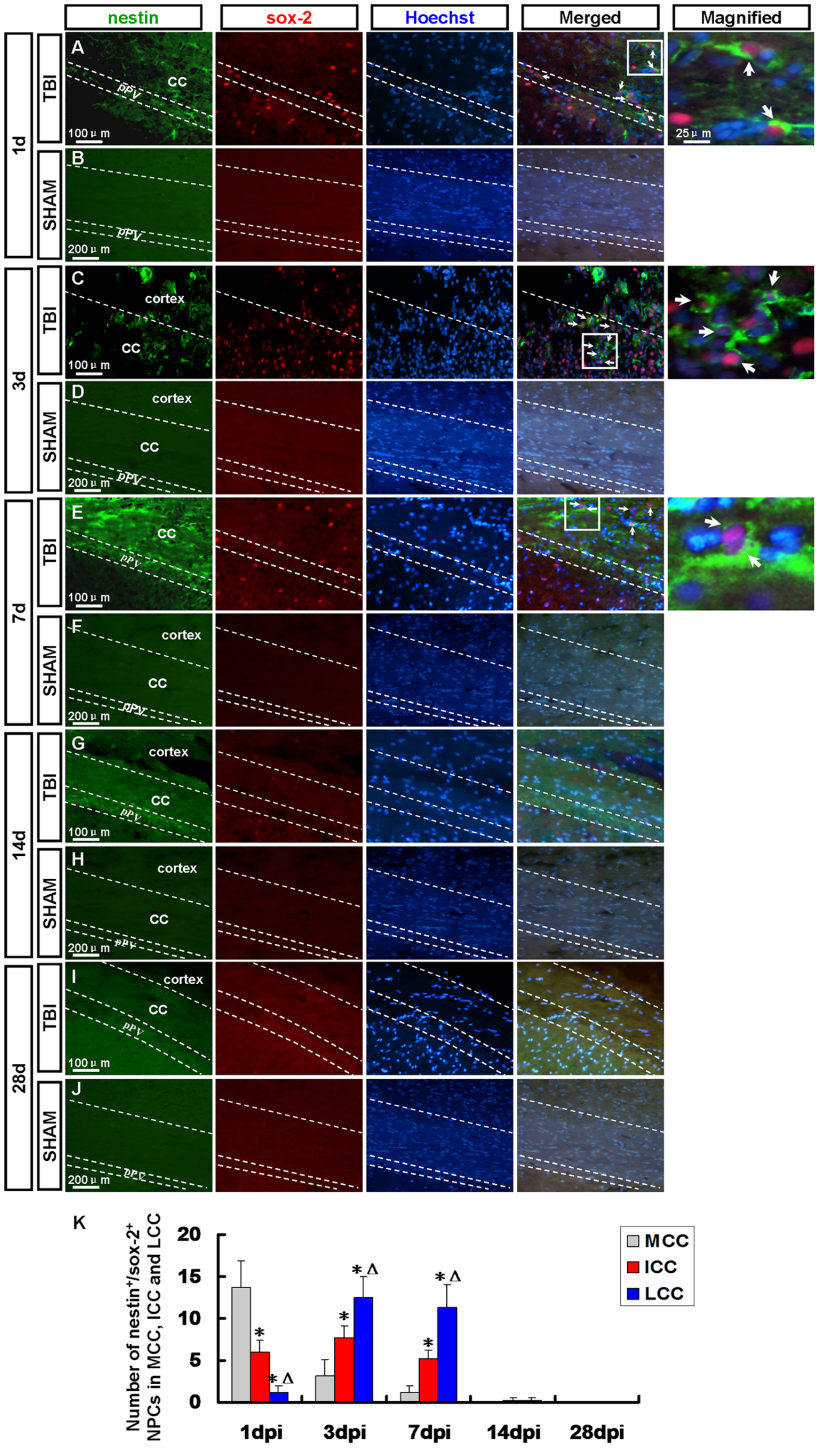
Endogenic nestin^+^/sox-2^+^ neural progenitor cells (NPCs) in the corpus callosum (CC) and posterior periventricle (pPV). NPCs were detected by nestin (green) and sox-2 (red) antibody. Arrows denote the nestin^+^/sox-2^+^ cells. (A) At 1 dpi many nestin^+^/sox-2^+^ NPCs were observed in the pPV and MCC where was adjacent to pPV. (C, E) At 3 and 7 dpi many nestin^+^/sox-2^+^ NPCs emerged in the ICC and LCC, especially in the latter where was adjacent to the peri-injured cortex. (G, I) Nestin^+^/sox-2^+^ NPCs were scarcely found in the pPV and CC at 14 and 28 dpi. (B, D, F, H, J) Nestin^+^/sox-2^+^ NPCs were not found in the sham group. (K) A statistic diagram for the number of nestin^+^/sox-2^+^ NPCs in the MCC, ICC and LCC at 1, 3, 7, 14 and 28 dpi. ^*^ *p*<0.05, vs. MCC; ^△^ *p*<0.05, vs. ICC.

Nestin^+^/sox-2^+^ NPCs were observed in the peri-injured cortex (Figure 2A, C, E, G and I), the ipsilateral CC and pPV (Figure 3A, C, E, G and I) at 1, 3, 7, 14 and 28 dpi. The number of NPCs in the peri-injured cortex peaked at 3 dpi then decreased (Figure 2K). Nestin^+^/sox-2^+^ NPCs were scarcely found at 28 dpi (Figure 2I). No positive cell was found in the sham group (Figure 2B, D, F, H and J).

At 1 dpi a robust increase in the number of nestin^+^/sox-2^+^ NPCs was observed in the pPV and medial area of corpus callosum (MCC) where was adjacent to pPV (Figure 3A, K), while it decreased at 3 and 7 dpi (Figure 3K) and many nestin^+^/sox-2^+^ NPCs emerged in the intermedial area of corpus callosum (ICC) and lateral area of corpus callosum (LCC) (Figure 3C, E and K), especially in the latter where was adjacent to the peri-injured cortex (Figure 3C, E and K). Nestin^+^/sox-2^+^ NPCs were scarcely found in the pPV and CC at 14 and 28 dpi (Figure 3G, I and K). No positive cell was found in the sham group (Figure 3B, D, F, H and J).

### 3.3 Activated GFAP^+^/sox-2^+^ RG-like Cells in Peri-injured Cortex

**Figure 4:**
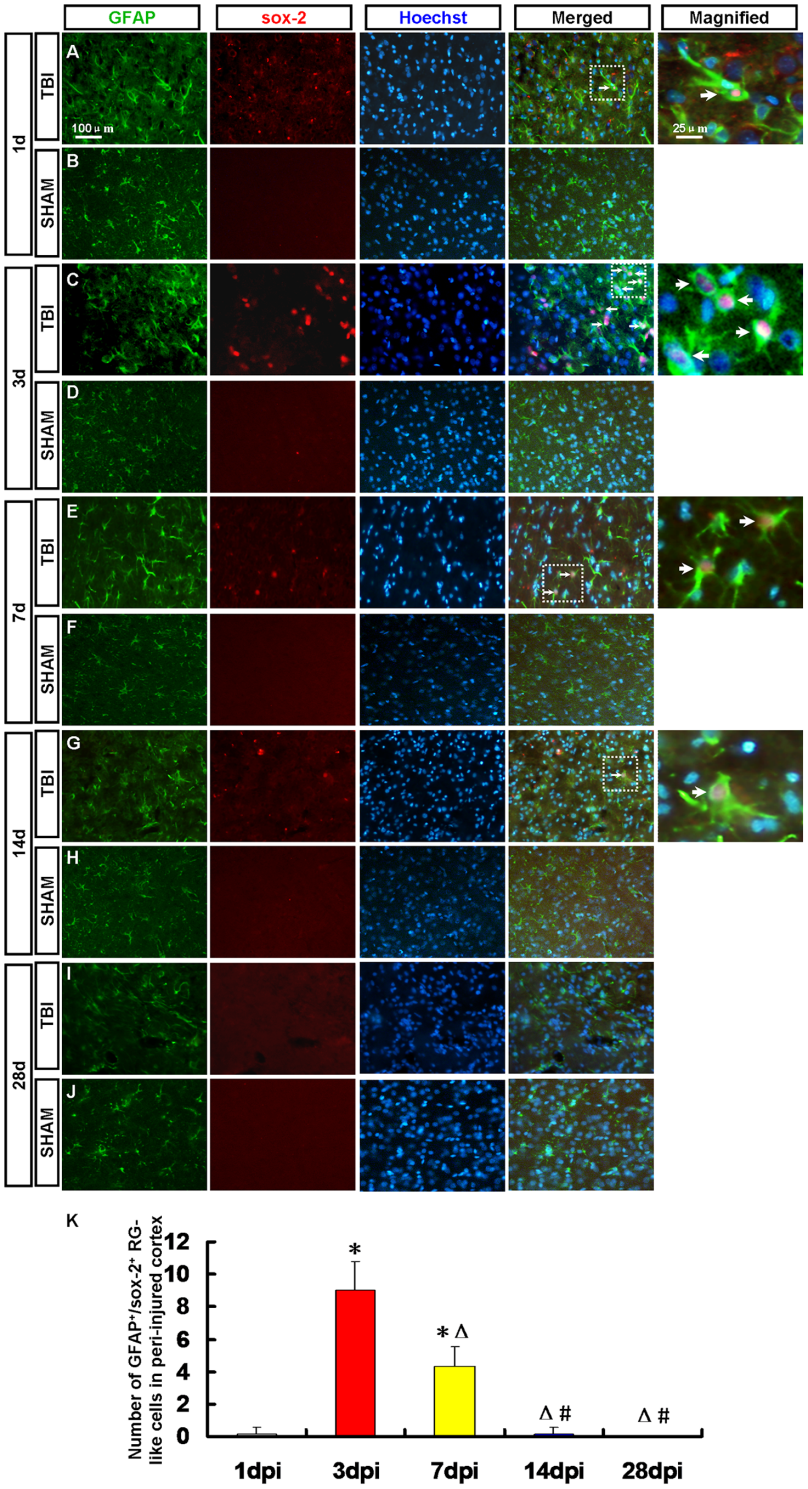
Activated GFAP^+^/sox-2^+^ radial glial (RG)-like cells in peri-injured cortex. RG-like cells were detected by GFAP (green) and sox-2 (red) antibody. Arrows denote the GFAP^+^/sox-2^+^ cells. (A, C, E, G, I) GFAP^+^/sox-2^+^ RG-like cells were observed in the peri-injured cortex at 1, 3, 7, 14 and 28 dpi. (B, D, F, H, J) GFAP^+^/sox-2^+^ RG-like cells were not found in the sham group. (K) A statistic diagram for the number of GFAP^+^/sox-2^+^ RG-like cells in the peri-injured cortex at 1, 3, 7, 14 and 28 dpi. The number of GFAP^+^/sox-2^+^ RG-like cells peaked at 3 dpi then decreased. ^*^ *p*<0.05, vs. 1 dpi group; ^△^ *p*<0.05, vs. 3 dpi group; ^#^ *p*<0.05 vs. 7 dpi group.

Radial glial cells (RGCs), a transient cell population present only in the developing CNS, provide an instructive scaffold for neuronal migration and function as a NPCs during cerebral cortical development [26], [27]. RGCs not only display astrocyte characteristics but also maintain features of stem cells [28]. Adult neurogenesis recapitulates the process of neuronal development in embryonic stages. GFAP and sox-2 double positive cells were detected to confirm whether actived RGCs emerged in the peri-injured cortex. GFAP^+^/sox-2^+^ radial glial (RG)-like cells were observed in the peri-injured cortex at 1, 3, 7, 14 and 28 dpi (Figure 4A, C, E, G and I). The number of RG-like cells in the peri-injured cortex peaked at 3 dpi then decreased (Figure 4K) in accord with change tendency of number of nestin/sox-2 positive NPCs. The number of GFAP^+^/sox-2^+^ RG-like cells was less than that of Nestin^+^/sox-2^+^ NPCs (Figure 2K and 4K). GFAP^+^/sox-2^+^ RG-like cells were not found in the sham group (Figure 4B, D, F, H and J).

**Figure 5:**
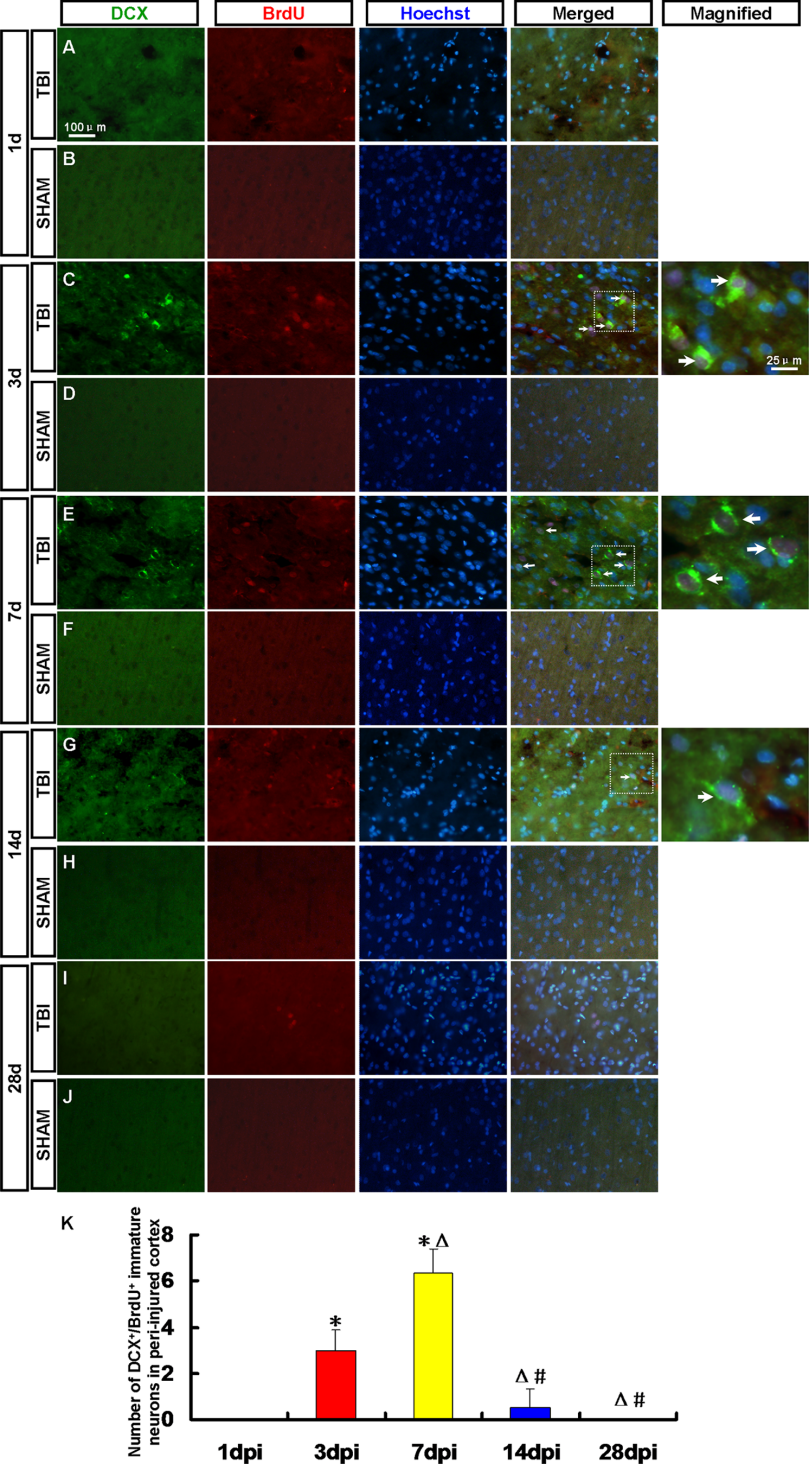
DCX^+^/BrdU^+^ newborn immature neurons in the peri-injured cortex. Newborn immature neurons in the peri-injured cortex were detected by DCX (green) and BrdU (red) antibody. Arrows denote the DCX^+^/BrdU^+^ cells**.** (A, C, E, G, I) DCX^+^/BrdU^+^ immature neurons were observed in the peri-injured cortex at 1, 3, 7, 14 and 28 dpi. (B, D, F, H, J) DCX^+^/BrdU^+^ immature neurons were not found in the sham group. (K) A statistic diagram for the number of DCX^+^/BrdU^+^ cells in the peri-injured cortex at 1, 3, 7, 14 and 28 dpi. ^*^ *p*<0.05, vs. 1 dpi group; ^△^ *p*<0.05, vs. 3 dpi group; ^#^ *p*<0.05 vs. 7 dpi group.

**Figure 6:**
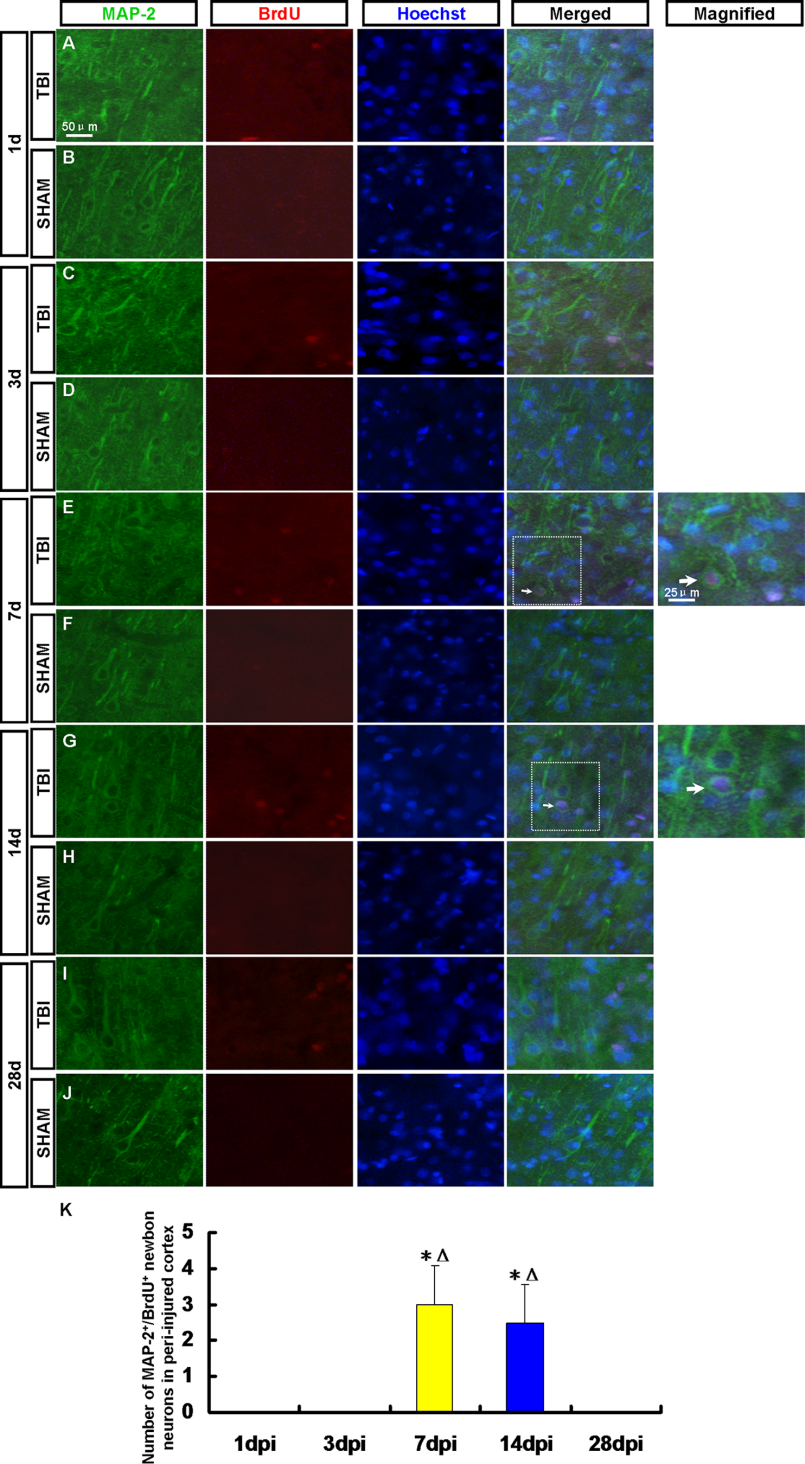
MAP-2^+^/BrdU^+^ newborn neurons in the peri-injured cortex. Newborn neurons in the peri-injured cortex were detected by MAP-2 (green) and BrdU (red) antibody. Arrows denote the MAP-2^+^/BrdU^+^ cells**.** (A, C, E, G, I) MAP-2^+^/BrdU^+^ neurons were observed in the peri-injured cortex at 1, 3, 7, 14 and 28 dpi. A few MAP-2^+^/BrdU^+^ cells were found in the peri-injured cortex only at 7 and 14 dpi. (B, D, F, H, J) MAP-2^+^/BrdU^+^ neurons were not found in the sham group. (K) A statistic diagram for the number of MAP-2^+^/BrdU^+^ cells in the peri-injured cortex at 1, 3, 7, 14 and 28 dpi. ^*^ *p*<0.05, vs. 1 dpi group; ^△^ *p*<0.05, vs. 3 dpi group.

**Figure 7:**
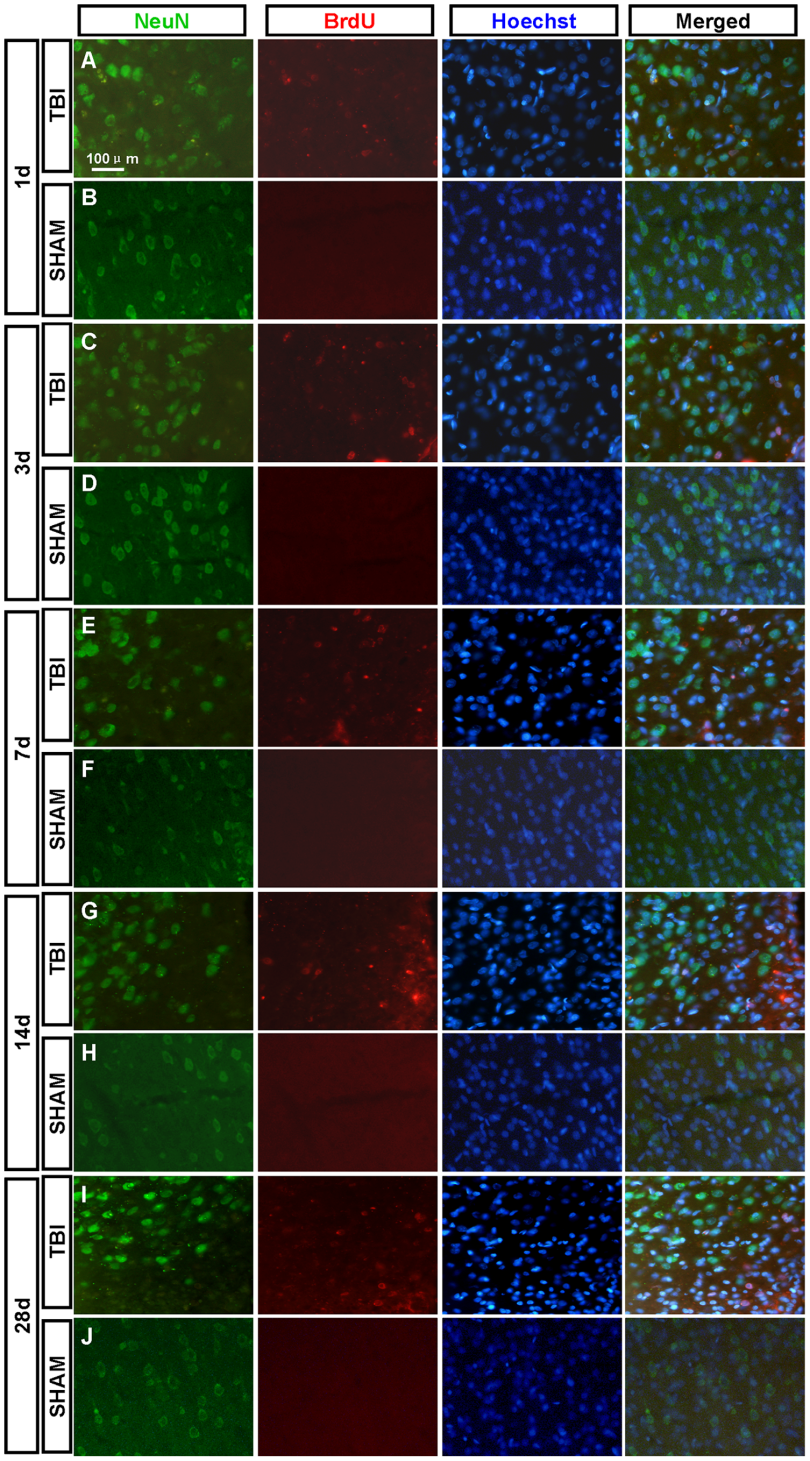
NeuN^+^/BrdU^+^ newborn mature neurons in the peri-injured cortex. Newborn mature neurons in the peri-injured cortex were detected by NeuN (green) and BrdU (red) antibody. NeuN^+^/BrdU^+^ newborn mature neurons were not found in the peri-injured cortex at 1, 3, 7, 14 and 28 dpi.

**Figure 8:**
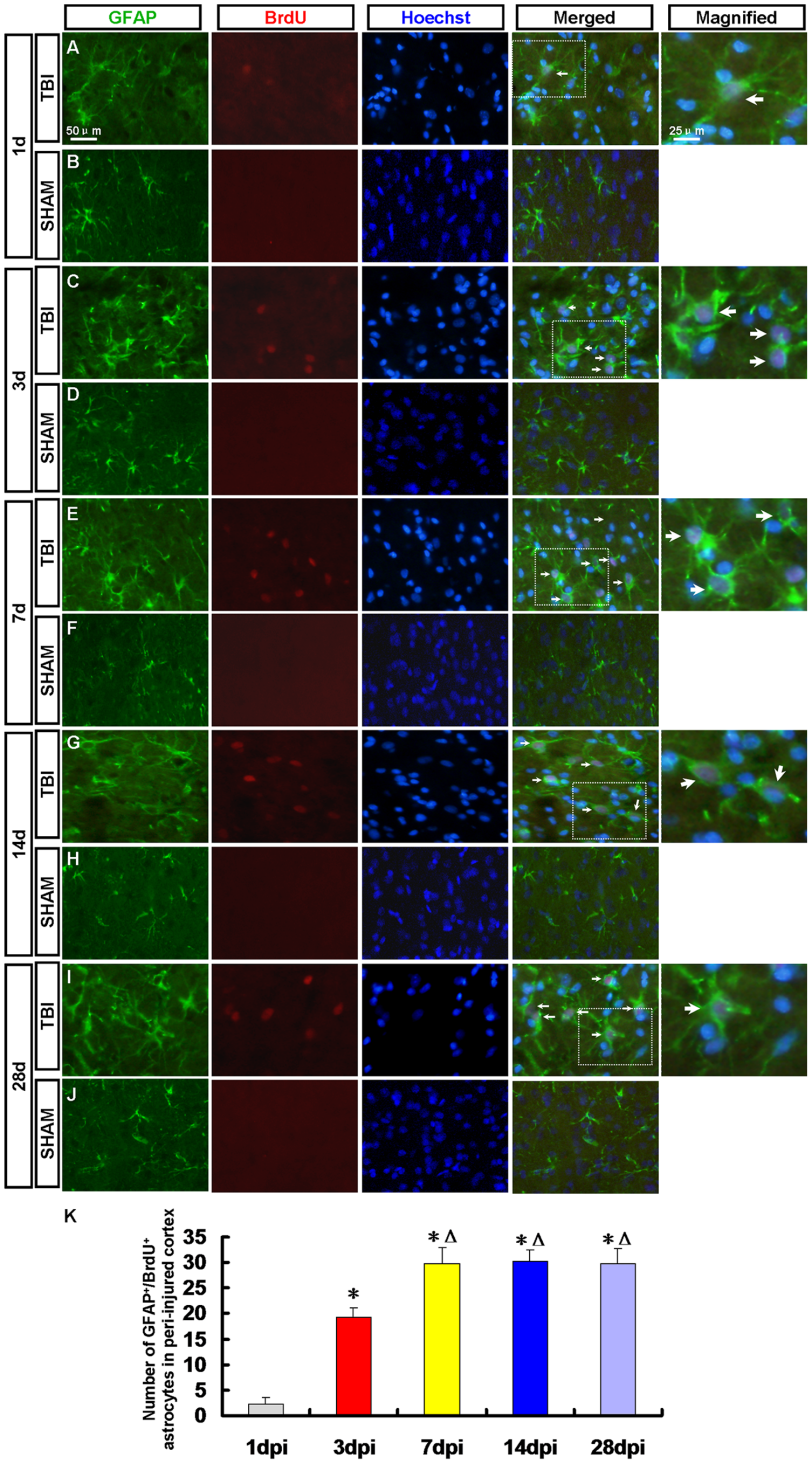
GFAP^+^/BrdU^+^ newborn astrocytes in the peri-injured cortex. Newborn astrocytes in the peri-injured cortex were detected by GFAP (green) and BrdU (red) antibody. Arrows denote the GFAP^+^/BrdU^+^ cells**.** (A, C, E, G, I) The GFAP^+^/BrdU^+^ astrocytes were found in peri-injured cortex at 1, 3, 7, 14 and 28 dpi. (B, D, F, H, J) GFAP^+^/BrdU^+^ astorcyets were not found in the sham group. (K) A statistic diagram for the number of GFAP^+^/BrdU^+^ cells in the peri-injured cortex at 1, 3, 7, 14 and 28 dpi. The number of GFAP^+^/BrdU^+^ cells peaked at 7 dpi then kept in a stable state. ^*^ *p*<0.05, vs. 1 dpi group; ^△^ *p*<0.05, vs. 3 dpi group.

**Figure 9:**
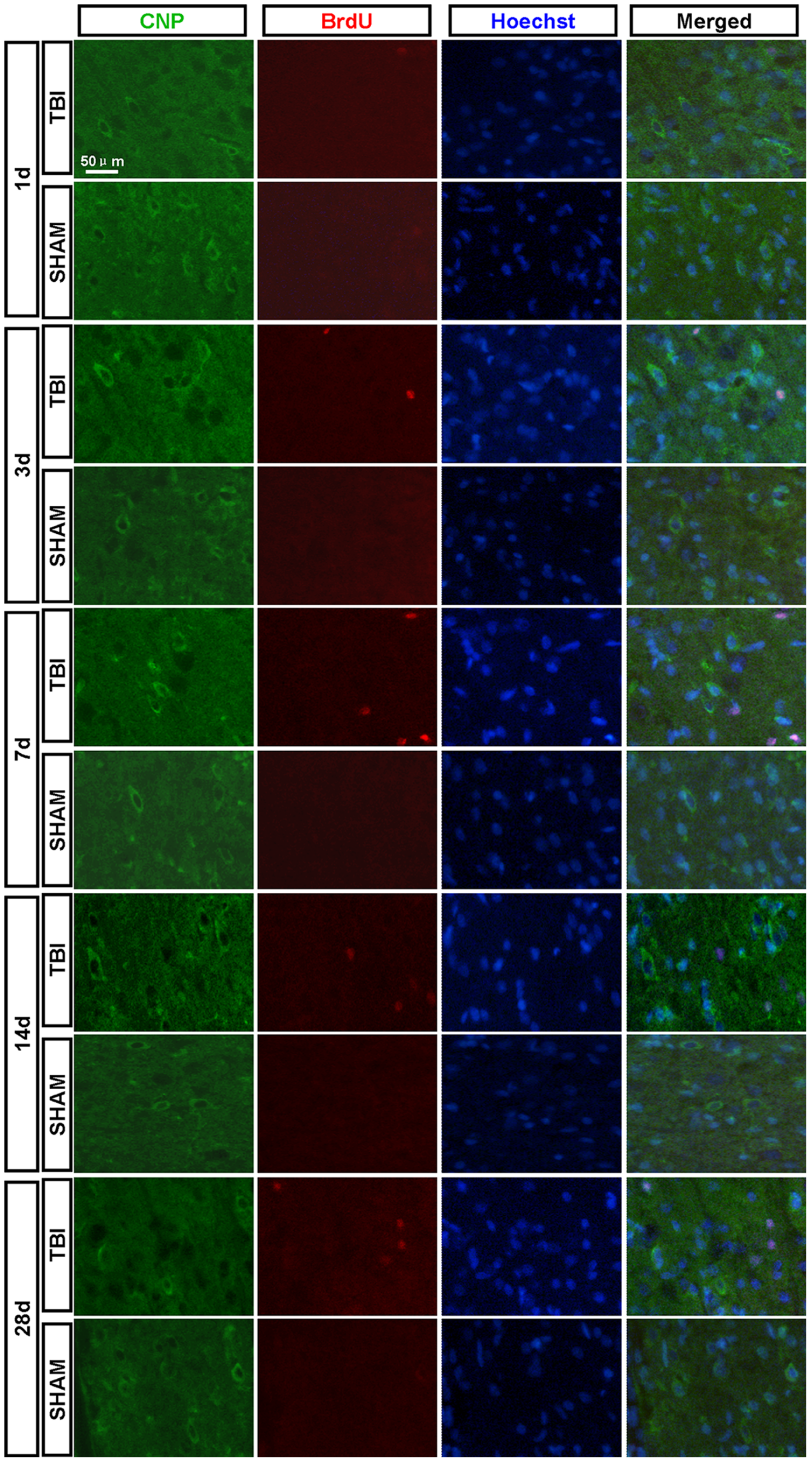
CNP^+^/BrdU^+^ newborn oligodendrocytes in the peri-injured cortex. Newborn oligodendrocytes in the peri-injured cortex were detected by CNP (green) and BrdU (red) antibody. CNP^+^/BrdU^+^ newborn oligodendrocytes were not found in the peri-injured cortex at 1, 3, 7, 14 and 28 dpi.

At 14 dpi, the number of nestin^+^/sox-2^+^ NPCs and GFAP^+^/sox-2^+^ RG-like cells decreased. Maybe some of them have differentiated into other types of cells or died. To determine the fate of the newborn cells in the peri-injured cortex after TBI, the newborn neurons, astrocytes and oligodendrocytes were detected in the peri-injured cortex by immunofluorescence (Figure 5, 6, 7, 8, 9).

### 3.4 Newborn Neurons in the Peri-injured Cortex

Neurons were detected by DCX/BrdU, MAP-2/BrdU and NeuN/BrdU staining respectively. DCX^+^/BrdU^+^ immature neurons were found in the peri-injured cortex at 3, 7 and 14 dpi (Figure 5C, E and G). More DCX^+^/BrdU^+^ cells were found in the peri-injured cortex at 7 dpi then decreased (Figure 5K). No DCX^+^/BrdU^+^ cell in the peri-injured cortex was found at 1 and 28 dpi (Figure 5A, I). A few MAP-2^+^/BrdU^+^ cells were found in the peri-injured cortex only at 7 and 14 dpi (Figure 6E, G). NeuN^+^/BrdU^+^ mature neuron has not been found in the peri-injured cortex at 1, 3, 7, 14 and 28 dpi (Figure 7A, C, E, G and I). MAP-2^+^/BrdU^+^ and NeuN^+^/BrdU^+^ cells were not found in the cortex of rats in the sham group (Figure 5, 6, 7-B, D, F, H and J).

### 3.5 Newborn Astrocytes in the Peri-injured Cortex

GFAP/BrdU staining was applied to confirm whether the NPCs in the peri-injured cortex differentiated into astrocytes. The GFAP^+^/BrdU^+^ astrocytes were found in peri-injured cortex at 1, 3, 7, 14, 28 dpi (Figure 8A, C, E, G and I) and peaked at 7 dpi then kept in a stable state (Figure 8K). No GFAP^+^/BrdU^+^ cell was found in the sham group (Figure 8B, D, F, H and J).

### 3.6 Newborn Oligodendrocytes in the Peri-injured Cortex

CNP^+^/BrdU^+^ cells were not found in peri-injured cortex and sham group (Figure 9).

### 3.7 The Percentage of NPCs, Newborn Neurons and Astrocytes of Newborn BrdU^+^ Cells

**Figure 10:**
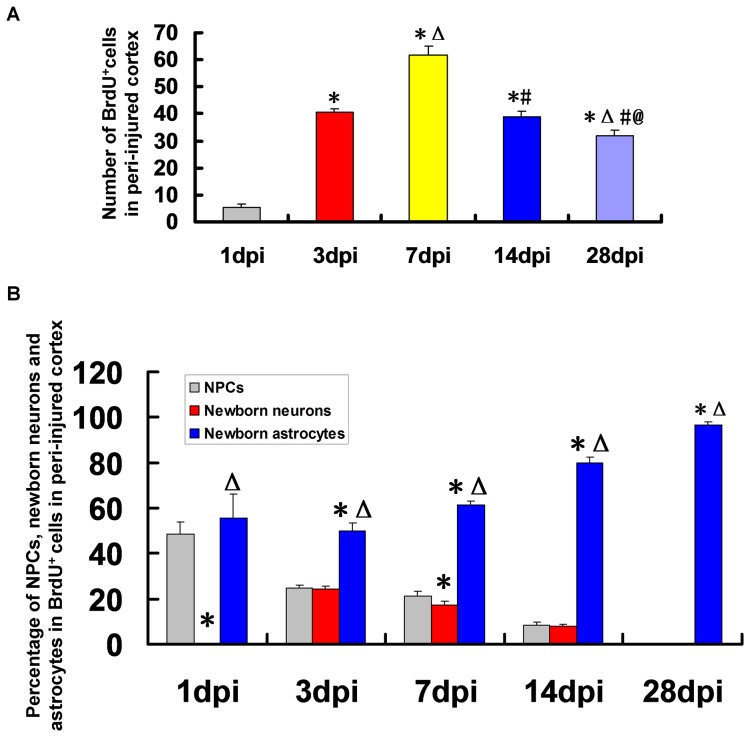
The percentage of neural progenitor cells (NPCs), newborn neurons and astrocytes in BrdU^+^ Cells. (A) A statistic diagram for the number of BrdU^+^ cells in the peri-injured cortex at 1, 3, 7, 14 and 28 dpi. The number of BrdU^+^ cells peaked at 7 dpi then decreased. (B) A statistic diagram for the percentage of NPCs, newborn neurons and astrocytes in BrdU^+^ Cells. The percentage of astrocytes (GFAP^+^/BrdU^+^) in newborn cells (BrdU^+^) was more than that of newborn neurons (DCX^+^/BrdU^+^+ MAP-2^+^/BrdU^+^+NeuN^+^/BrdU^+^) or NPCs (nestin^+^/sox-2^+^) in the corresponding time point. At 28 dpi, almost all newborn cells were astrocytes. ^*^ *p*<0.05, vs. 1 dpi group; ^△^ *p*<0.05, vs. 3 dpi group; ^#^ *p*<0.05, vs. 7 dpi group; ^@^ *p*<0.05, vs. 14 dpi group.

BrdU^+^ cells were found in peri-injured cortex at 1, 3, 7, 14, 28 dpi (Figure 5, 6, 7, 8, 9) and peaked at 7 dpi then decreased (Figure 10A). The results indicated that some newborn cells died in the following days. Newborn BrdU^+^ cells in peri-injured cortex were mainly composed of NPCs, newborn differentiated cells and reactive gliosis cells. In the corresponding time point, the percentage of astrocytes (GFAP^+^/BrdU^+^) in newborn cells was more than that of newborn neurons (DCX^+^/BrdU^+^+ MAP-2^+^/BrdU^+^+NeuN^+^/BrdU^+^) or NPCs (nestin^+^/sox-2^+^) (Figure 10B). At 28 dpi, almost all newborn cells were astrocytes (Figure 10B).

## Discussion

TBI includes skull fracture, acute cerebral vascular damage in the acute phase, primary mechanical injury to the brain tissue, and these traumatic events result in a series of cellular and molecular reactions that induce secondary brain injury. All these lead to neuronal loss in TBI. With the lack of effective therapy to repair injured brain tissue, has motivated researchers to focus on the use of endogenous stem cells. In contrast to the belief that the cells of the adult mammalian brain cannot be regenerated after injury, recent findings have indicated not only the presence of stem cells in the brain throughout life but also that neurogenesis is an ongoing process into adulthood [29], [30]. There are two areas in the mammalian brain, SVZ and SGZ, where neurogenesis occurs throughout adulthood. The newborn neurons in the SGZ become incorporated into the normal hippocampal circuitry [31], whereas neuroblasts generated in the SVZ migrate toward the olfactory bulb (OB) where they differentiate into neurons [32], [33]. Similar studies have established that neurogenesis also occur in the SGZ and SVZ of adult primates, including humans [12], [34]. Enhancement of neurogenesis in the two zones is confirmed in some rodent models with brain injury [15]–[18]. Whether adult neurogenesis is constrained only in the two canonical zones, or could occur also in other CNS areas after brain injury is still an open question [19].

Previous study reported that neural stem cells had been isolated in vitro only from the damaged rat cerebral cortex at 3 dpi, corresponding to a peak in nestin expression in peri-injured cortex [22]. In our present study, we investigated endogenous neural regeneration in the peri-injured cortex after TBI in vivo. At 1, 3, 7 and 14 dpi nestin^+^/sox-2^+^ NPCs were found in the peri-injured cortex and peaked at 3 dpi. The NPCs maybe immigrate from other zones or be the resident cells.

At 1 dpi a dramatic increase in the number of nestin^+^/sox-2^+^ NPCs were observed in the pPV and MCC where was adjacent to pPV, while it decreased at 3 and 7 dpi and many nestin^+^/sox-2^+^ NPCs emerged in the ICC and LCC, especially in the latter where was adjacent to the peri-injured cortex. Recent studies have demonstrated that the pPV, which is the posterior part of the LV, also retains proliferating neural progenitors [24] and they could migrate into the hippocampal CA1 region in rats after ischemic injury [25]. Our data showed the NPCs from pPV could migrate into the peri-injured cortex through CC after TBI.

Magavi and his colleagues reported that the latent NPCs in cortex have been activated after brain injury [35]. The latent NPCs belong to which type cells remains to be confirmed. It has been previously suggested that mature astrocytes could transform into transitional RGCs in the adult mouse neocortex [36]. RGCs not only display astrocyte characteristics but also maintain features of stem cells. The present study GFAP^+^/sox-2^+^ RG-like cells in the peri-injured cortex were found. The number of RG-like cells was less than that of nestin^+^/sox-2^+^ NPCs at 3 and 7 dpi.

The number of GFAP^+^/sox-2^+^ RG-like cells in the peri-injured cortex peaked at 3 dpi then decreased in accord with change tendency of number of nestin^+^/sox-2^+^ NPCs. Maybe some of them have differentiated into other types of cells or died in the following days. In the present study, the newborn neurons, astrocytes and oligodendrocytes were detected in the peri-injured cortex by immunofluorescence to determine the fate of the newborn cells in the peri-injured cortex after TBI.

Neurons were detected by DCX/BrdU, MAP-2/BrdU and NeuN/BrdU staining respectively. At 3, 7 and 14 dpi DCX^+^/BrdU^+^ immature neurons were found in the peri-injured cortex. More DCX^+^/BrdU^+^ cells were found in the peri-injured cortex at 7dpi then decreased. No DCX^+^/BrdU^+^ cell was found at 1 and 28 dpi. A few MAP-2^+^/BrdU^+^ cells were found in the peri-injured cortex only at 7 and 14 dpi. NeuN^+^/BrdU^+^ mature neurons were not found in the peri-injured cortex at 1, 3, 7, 14 and 28 dpi. The results of our study showed that the newborn cells in peri-injured cortex of adult rat after TBI could differentiate into immature neurons, but the latter failed to grow up to mature neurons. Maybe they did not survive due to apoptosis [37]. So we need to explore the ways of preventing the newborn neurons from apoptosis to improve the regeneration in the further study. Newborn cells are sensitive to pathological niche and most of them die, and others become astrocytes or oligodendrocytes [38], [39]. In our study, newborn BrdU^+^ cells peaked at 7 dpi then decreased which indicated some newborn cells died in the following days. No CNP^+^/BrdU^+^ oligodendrocytes but the GFAP^+^/BrdU^+^ astrocytes were found in peri-injured cortex at 1, 3, 7, 14, 28 dpi and peaked at 7 dpi then kept in a stable state. Newborn BrdU^+^ cells in peri-injured cortex were mainly composed of NPCs, newborn differentiated cells and reactive gliosis cells. In the corresponding time point, the percentage of GFAP^+^/BrdU^+^ astrocytes in newborn cells was more than that of newborn neurons or NPCs. The results indicate that most of NPCs maybe differentiate into glial cells. Obviously some of GFAP^+^/BrdU^+^ astrocytes also originate in the local reactive gliosis. The mechanisms involving them need to be further explored. The neurogenesis was not found in the normal cortex.

In conclusion, our research indicates that the neurogenesis happens in the peri-injured cortex of adult rats after TBI, the NPCs can differentiate into neurons and astrocytes, but no newborn mature neuron is found. These findings suggest that endogenous neurogenesis and inhibiting apoptosis of newborn cells maybe act as potential strategies to improve the consequences of TBI.
